# Oscillatory interaction between amygdala and hippocampus coordinates behavioral modulation based on reward expectation

**DOI:** 10.3389/fnbeh.2013.00177

**Published:** 2013-12-03

**Authors:** Satoshi Terada, Susumu Takahashi, Yoshio Sakurai

**Affiliations:** ^1^Department of Psychology, Graduate School of Letters, Kyoto UniversityKyoto, Japan; ^2^Laboratory of Neural Circuitry, Graduate School of Brain Science, Doshisha UniversityKizugawa, Japan

**Keywords:** reward expectation, behavioral modulation, oscillation synchrony, amygdala, hippocampus

## Abstract

The aim of this study is to examine how the amygdala and hippocampus interact for behavioral performance modulated by different Reward-expectations (REs). We simultaneously recorded neuronal spikes and local field potential from the basolateral amygdala and hippocampal CA1 while rats were performing a light-side discrimination task with different expectations of a high or low probability of reward delivery. Here, we report the following results. First, the rats actually modulated their behavioral performance on their expectations of a high or low probability of reward. Second, we found more neurons related to RE in the amygdala and more neurons related to task performance in the hippocampus. Third, a prominent increase in the coherence of high-frequency oscillations (HFOs) (90–150 Hz) between the amygdala and the hippocampus was present during high RE. Fourth, coherent HFOs during inter-trial intervals and theta coherence during trials had significant correlations with the behavioral goal-selection time. Finally, cross-frequency couplings of LFPs within and across the amygdala and hippocampus occurred during ITI. These results suggest that the amygdala and hippocampus have different functional roles in the present task with different REs, and the distinctive band of coherence between the amygdala and the hippocampus contributes to behavioral modulation on the basis of REs. We propose that the amygdala influences firing rates and the strength of synchronization of hippocampal neurons through coherent oscillation, which is a part of the mechanism of how reward expectations modulate goal-directed behavior.

## Introduction

The expectation of a future reward powerfully modulates goal-directed behavior. Animals and humans behave quickly and eagerly when they predict valuable and highly probable rewards. Although several brain regions play important and different roles in this prediction behavior (Corbit et al., 2001; Ostlund and Balleine, 2007; Johnson et al., 2009; Holmes et al., 2010), interaction among these regions is also essential (Holland and Gallagher, 2004; Schoenbaum and Roesch, 2005; Ito et al., 2008; Shiflett and Balleine, 2010). Little is known, however, about the mechanism of regional interaction in modulating behaviors on the basis of reward expectation (RE). Brain rhythms, especially oscillations, across a wide range of frequencies (Buzsaki, 2006; Womelsdorf et al., 2007; Siegel et al., 2012) are likely to contribute to such interaction among the distributed neural circuits. Actually, some previous studies have revealed that brain oscillations underlie various behavioral and emotional functions (Bauer et al., 2007; Popescu et al., 2009; van der Meer and Redish, 2011). Most of the previous studies, however, were based on recording only from reward-related or behavior-related regions and few investigated the role of oscillations in the neural communication between different brain regions for behavioral modulation on RE.

In the present study, we focused on oscillatory interactions between the amygdala and the hippocampus. The amygdala is a well-known reward-related brain region and is critical for RE (Blundell et al., 2001; Savage and Ramos, 2009; Morrison and Salzman, 2010). The hippocampus, on the other hand, is not critical for RE (Ramirez and Savage, 2007) but has important roles in the learning and performance of behaviors. Also, hippocampal oscillations are correlated with the improvement of task-related behavioral performance (Rutishauser et al., 2010; Ahmed and Mehta, 2012). The hippocampal functions for behavioral performance have been shown to be affected by reward (Holscher et al., 2003; Tabuchi et al., 2003; Singer and Frank, 2009). Moreover, the interaction between the amygdala and the hippocampus is thought to be involved in the emotional modulation of behavior. For example, a recent disconnection study indicated that the amygdala-hippocampus interaction is essential for context-induced cocaine-seeking behavior (Wells et al., 2011). In Pavlovian fear conditioning, these brain regions were proposed to communicate with each other via the coherent theta oscillations (Pape et al., 2005; Lesting et al., 2011). Electrical stimulation of the amygdala was shown to be able to produce alterations in the firing properties of hippocampal place cells (Kim et al., 2012). Despite these previous observations, few studies have directly specified the role of coherent oscillations between the amygdala and the hippocampus in modulating behaviors based on RE.

In this study, we simultaneously recorded neuronal spikes and local field potential (LFP) from the amygdala and hippocampus while rats were performing a reward-probability-biased discrimination task. We hypothesized that the amygdala represents the RE for different probabilities of reward and the hippocampus represents the appropriate behavioral performance required for the task. The activities of the amygdala and hippocampus are expected to exhibit synchrony in oscillations when the REs modulate the behavioral performance of the task.

## Materials and methods

### Subjects

Eight male Wistar albino rats (Shimizu Laboratory Supplies, Kyoto, Japan), each weighing 400–480 g at the time of the experiment and housed in a 25 × 15 × 24 cm cage, were used as experimental subjects. All rats were handled extensively, provided with a sufficient amount of lab chow 1–3 h after each daily training or recording session to maintain approximately 85% of their ad libitum weight during daily training or recording sessions, and allowed free access to water. They were exposed to light between 08:00 and 21:00 h each day. All experiments were conducted between 10:00 and 20:00 h in accordance with the guidelines presented in Guidelines for Care and Use of Laboratory Animals at Kyoto University (2007) and with the approval of the Animal Research Committee of Kyoto University.

### Apparatus

In a dim, sound-attenuated, electrically shielded box (Japan Shield Enclosure, Osaka, Japan), rats were trained in a behavioral task in a 22 × 32 × 45 cm operant chamber (Ohara Ika, Tokyo, Japan). One wall of the chamber had three 15-mm-diameter illuminated sensor holes in the horizontal direction 60 mm above the floor to detect the nose-poke behavior of rats. Access to the left and right holes was controlled using a guillotine door immediately in front of each hole. A food dispenser behind the wall delivered 25 mg food pellets to a food magazine located at the center of the wall and 10 mm above the floor. The dispenser delivered pellets with an intermittent low buzzer tone (reward tone). Another buzzer was located behind the food dispenser to present a continuous high buzzer tone (error tone) when the rats made erroneous responses. Visual stimuli were presented on the left or right wall using a light-emitting diode (LED). Auditory stimuli consisting of two pure tones (2 or 10 kHz) of approximately 70 dB SPL were presented via a loudspeaker (15 cm in diameter) set 30 cm above the top of the operant chamber. The task was controlled and the behavioral data were recorded using a personal computer (NEC, Tokyo, Japan). Each rat's behavior was monitored using a video camera (Sony, Tokyo, Japan), and the extracellular neuronal activity from 24 channels was simultaneously amplified using a multichannel amplifier system (Nihon Kohden, Kyoto, Japan) and stored in a custom-made personal computer.

### Light-side discrimination task

**Figure 1:**
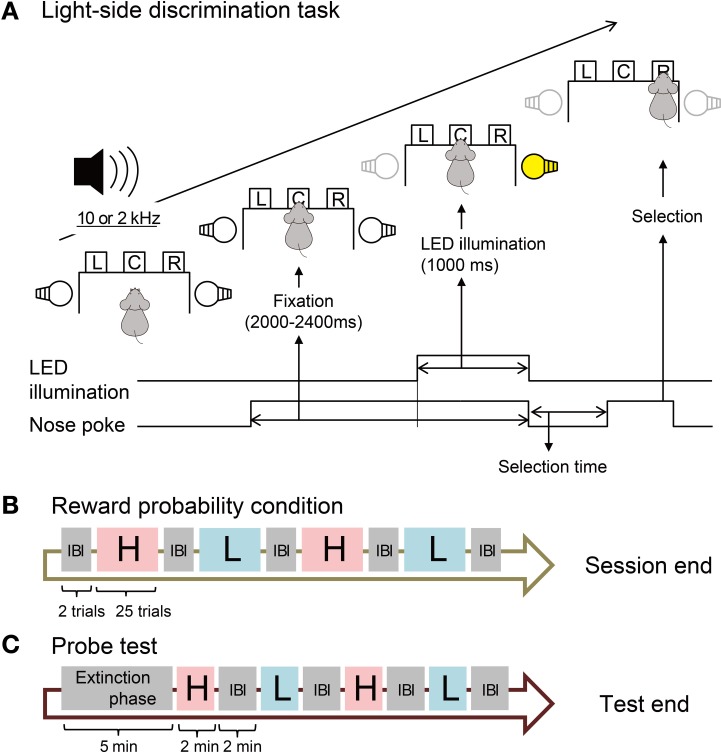
**Design of behavioral task. (A)** Procedure of the light-side discrimination task. One of two pure tones (2 and 10 kHz) is constantly presented during the trial. The operant chamber is equipped with left (L), center (C), and right (R) sensor holes for nose-poke behavior. The bottom lines and arrows show the time course of events in one trial. **(B)** Sequence of blocks with different reward probability conditions. In each block of 25 trials, the reward probability is fixed to high (H, 80%) or low (L, 20%). The rats are informed of the high or low probability of the reward by one of the two pure tones. No tone is presented in inter-block intervals (IBI). **(C)** Sequence of blocks in the probe test. One of the two pure tones is presented in each block but rats obtain no reward. No tone is presented during the extinction phase.

Rats were trained to perform a reward-probability-biased light-side discrimination task (Figure 1A). Each trial started with the rat holding its nose in the central hole for 1000–1400 ms. Then the left or right LED was illuminated for 1000 ms (fixation phase) during which the rat continued to keep its nose in the hole. After turning off the LED illumination, the rat was required to poke its nose into the left or right hole illuminated by the LED (selection phase). The correct response was always indicated by presentation of the reward tone for 1000 ms. The probability of delivery of the reward (pellet) accompanying the reward tone was determined by the following reward probability (RP) conditions. Each trial was separated by an inter-trial interval (ITI) of 5000 ms.

### Reward probability conditions

In each session, we divided the total of 100 trials into 4 blocks, each of which consisted of 25 trials with one RP condition (Figure 1B). We prepared high (H) and low (L) RPs during which 10 and 2 kHz pure tones were constantly presented, respectively (Figure 1A). Under the H-RP condition, the probability of reward delivery was 80% for all correct trials. Only reward tones were presented in the remaining 20% of correct trials. Under the L-RP condition, the probability of reward delivery was 20% for all correct trials and in the remaining 80% of correct trials, only reward tones were presented. A session contained two blocks with the H-RP condition and two blocks with the L-RP condition. The order of blocks with H- or L-RPs in one session was H–L–H–L, L–H–L–H, H–L–L–H, or L–H–H–L, and was randomly varied day by day. We carried out two trials in an inter-block interval (IBI), during which neither the high nor the low tone was presented and the rats obtained no reward in all trials.

### Probe test

After all recording sessions under the above conditions, we implemented a probe test session for each rat to confirm that the rats actually predicted the high and low probabilities of reward delivery using the presented high and low tones (Figure 1C). No reward was delivered in all trials throughout the session. In the extinction phase (5 min), no tone was presented and the rats were able to start each trial with no ITI. Following the extinction phase, probe test blocks were implemented. One block and the IBI were 2 min each. The procedure of the probe test was identical to that of the extinction phase but the high or low tone was constantly presented during each block. The probe test blocks were H–L–H–L or L–H–L–H in the session.

### Data analysis of behavior

To investigate whether the rats modulated their behavior in accordance with their REs, selection times and correct rates under the different H- and L-RP conditions were analyzed. The selection time was defined as the time taken by the rats to select the right or left hole in the selection phase (Figure 1A). To confirm that the rats predicted the high or low probability of reward delivery on the basis of the high or low tone, the number of trials started by the rats under the different RP conditions indicated by the high and low tones in the probe test was analyzed. ANOVA was used to compare the differences between the reward probabilities (H-RP condition and L-RP condition). To exclude position bias, we added the cue direction (right and left) for the nose poke as the second variable to exclude position bias in the analysis of the selection time.

### Electrode construction

Neuronal recording was performed with tetrodes (Wilson and McNaughton, 1993), each of which was composed of four tungsten microwires (12.5 micron in diameter; California Fine Wire, Grover Beach, CA). The four microwires were mounted in a 33 gauge stainless-steel cannula (Small Parts, Miami, FL) with 500 micron of the tip protruding. The tips were cut at right angles with sharp surgical scissors. The tip impedance was approximately 400 kΩ at 1 kHz. Two or three cannulas with tetrodes were attached in a row to construct an array of tetrodes, with a center-to-center spacing of 500 micron between the cannulas. The array of tetrodes was mounted on a microdrive assembly (McNaughton et al., 1989; Sakurai, 1993, 1994, 1996, 2002; Sakurai and Takahashi, 2006) designed to allow fine movements of the cannulas with tetrodes and stable recording of multineuronal activity for extended periods.

### Implantation surgery

Details of surgery and data collection have been described previously (Takahashi and Sakurai, 2009a,b; Sakurai and Takahashi, 2013). After the completion of behavioral training, each rat was anesthetized with sodium pentobarbital (40 mg/kg) in preparation for the attachment of two microdrives, each of which contained two or three tetrodes, to the skull surface. After holes were drilled into the skull for electrode implantation into the basolateral amygdala (−2.28 mm from the bregma and 5.0 from the midline) and the hippocampal CA1 (−3.5 mm from the bregma and 2.5 mm from the midline), the tips of the tetrodes were implanted into the brain to a depth of approximately 4000 micron prior to the basolateral amygdala and 2000 micron prior to the hippocampal CA1. The reason for the small depth of the tetrodes targeting the amygdala was to decrease the damage to the brain and to obtain stable and long-term recordings. The craniotomy was filled with white petrolatum to a level slightly above the exit of the tetrodes from the skull surface. After the supports of the microdrives and cannulas were coated with a thin film of white petrolatum, the entire assembly was embedded in dental cement on the skull surface. A recovery period of about a week was assigned after surgery. We performed microdrive lowering of the tetrodes post-surgery to obtain stable long-term recordings as in the previous studies (Takahashi and Sakurai, 2009a,b; Sakurai and Takahashi, 2013).

### Data collection

Brain activity data were recorded and stored on a hard disk of a personal computer at a 20 kHz sampling rate while the rats were performing the behavioral task. Head stages containing 24 field-effect transistors (Toshiba, Tokyo, Japan) that had been set as source followers were used to connect a 24-channel plastic connector that had been cemented to the animal's head with preamplifiers. The output signals of the preamplifiers, which contained differential operational amplifiers, were transmitted to the main amplifiers in which amplified analog signals were band-pass filtered at 0.5–10 kHz for spiking activities and 0.08–300 Hz for LFP activity.

### Spike sorting for data analysis

Details of spike sorting were reported previously (Takahashi et al., 2003a,b; Sakurai and Takahashi, 2006). Recorded spike trains were sorted to isolate individual neuronal activities by a method of independent component analysis (ICA) and k-means clustering called ICsort (Takahashi et al., 2003a,b). After spike sorting, the isolation quality was visually inspected in the 1st to 3rd principal components feature spaces.

### Analysis of task-related spiking activity of neurons

Significant difference in the spiking activity of all neurons were confirmed by a statistical test (confidence limit, Abeles, 1982). The procedure of calculating the upper and lower confidence limits (Sakurai et al., 2004) is as the following. Under the null hypothesis that the spiking activity arises at a constant average rate and that this firing rate is independent of the history of the neuron firing and other events (e.g., the task phases and the RP conditions), including the firing of other neurons expected to be independent Poisson processes, the firing rate in each bin (50 ms) (*n*) is expected with the average firing rate during whole periods of the tasks, the bin size and the number of events. Under these assumptions, the probability of finding *m* spikes in the bin is given by the Poisson formula:

$$P\left(m,n\right)=\frac{e^{-n}n^{m}}{m!}$$

Then the lower confidence limit is set at one less than the smallest *m* for:

$$\sum_{i=0}^{m}P\left(i,n\right)>0.005$$

and the upper confidence limit is the smallest *m* for

$$\sum_{i=0}^{m}P\left(i,n\right)>0.995$$

The spiking activity is defined as significant task-related activity when more than two successive bins (100 ms) are above twice the band between the upper and lower confidence limits. If a neuron showed statistically significant differences in its activity between H- and L-RP conditions and/or between nose-pokes to the left and right holes, the neuron was designated as a task-related one. Then we classified each of the task-related neurons as reward-expectation (RE) neurons and/or task-performing (TP) neurons. The former were defined as neurons that show a statistically significant difference in the spiking activity between the different RP conditions, and the latter were defined as neurons that show significantly different spiking activity between left and right nose-pokes on the basis of discriminative cues provided by LED illumination.

### Analysis of LFP

Coherence and averaged power spectral densities (PSDs) of LFP were calculated by multitaper Fourier analysis by applying the Chronux toolbox (http://www.chronux.org) (Mitra and Bokil, 2008; Bokil et al., 2010). To obtain fine time dynamics of the coherence of LFP between the amygdala and the hippocampus, coherograms were computed for each LFP pair per session (van der Meer and Redish, 2011). Coherograms estimate the coherency C between the two LFP power spectra *X* and *Y* for each frequency *f* as

$$C_{XY}(f)=\frac{S_{XY}(f)}{\sqrt{S_{XX}(f)S_{YY}(f)}}$$

with

$$S_{XY}(f)=\frac{1}{K}\sum_{k\;=\;1}^{K}x_{k}(f)y_{k}(f),$$

where *x*_*k*_ and *y*_*k*_ are the two LFP spectra as follows:

$$x_{k}(f)=\sum_{t=1}^{T}w_{t}(k)x_{t}e^{-2\pi ift}.$$

We used the Chronux cohgramc function and mtspectrumc function with the following parameters: window size, 0.5 s; time step, 100 ms; five or ten tapers. Before the analysis, all LFP data were removed of their direct current offsets, slowly changing components, and 50/60 Hz line noise by using the locdetrend function and rmlinese function. The frequencies of each LFP were classified into theta band (5–10 Hz), gamma band (30–80 Hz), or high-frequency band (90–150 Hz) oscillations (Fujisawa and Buzsaki, 2011; van der Meer and Redish, 2011; Buzsaki and Silva, 2012; Tort et al., 2013).

To quantify the amplitude modulation by phases, we calculated the modulation index (MI) in accordance with the procedure described in Tort et al. (2008). This index can detect cross-frequency coupling (phase-amplitude coupling; PAC) between two frequency ranges of interest (e.g., HFO/gamma and theta). To test for significant differences among cross-frequency couplings within and across the regions, we compared the peak MIs by three-way ANOVA with the following factors: REs (H-RP condition and L-RP condition), task periods (ITI and the trial period), and pairs for PAC (PACs within the amygdala, PACs within the hippocampus, amygdala theta phase—hippocampal HFO amplitude, and hippocampal theta phase—amygdala HFO amplitude).

### Correlation between coherence and behavioral performance

We calculated the Pearson product-moment correlation coefficient (Pearson's *r*) between LFP coherence in the ITI or the trial and the selection time of behavioral performance following the ITI. To avoid spurious correlation, we calculated Pearson's *r* under H- and L-RP conditions respectively. Moreover, to exclude correlations that arose by chance (sham correlations), we recalculated Pearson's *r* using selection times in the next trials (shifted trials). We had also calculated the Spearman's rank correlation coefficient, but the results were similar to those obtained by Pearson's *r*.

### Histology

After the experiment was conducted, the rats were anesthetized deeply with an overdose of sodium pentobarbital (120 mg/kg) before being perfused and fixed with 10% buffered formalin solution. After the brain was sectioned at 50 μm intervals, the locations of the electrode tips and tracks in the brains were identified with the aid of a stereotaxic atlas (Paxinos and Watson, 2009).

## Results

### Behavior

**Figure 2:**
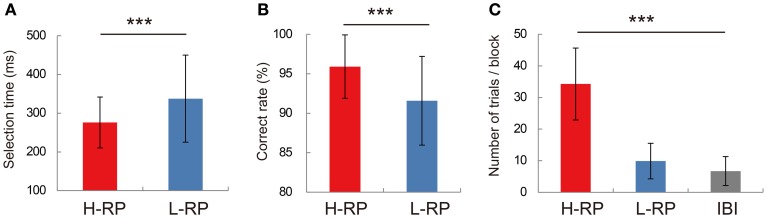
**Behavioral performance in recording sessions. (A)** Mean selection times under the H-RP and L-RP conditions in all sessions. Error bars show standard deviations. **(B)** Mean correct rates under the H-RP and L-RP conditions in all sessions. **(C)** Mean numbers of trials the rats performed in blocks and IBI in the probe test. Asterisks indicate significant differences of ^***^p < 0.005.

We used data from 58 sessions to test the behavior of rats by ANOVA. The rats showed different behavioral performance under different RP conditions (Figure 2). Under the H-RP condition, all rats responded with nose-pokes significantly faster than under the L-RP condition [Figure 2A; two-way ANOVA, *F*_(1, 5194)_ = 593.67, *p* < 0.001]. We have confirmed the significantly different selection times between RP conditions for each rat. Also, the rats under the H-RP condition performed the task more accurately than those under the L-RP condition [Figure 2B; ANOVA, *F*_(1, 84)_ = 26.8, *p* < 0.001]. In the probe test during which no reward was delivered, the rats repeated significantly more trials when the high tone indicating H-RP was presented than when the low tone indicating L-RP was presented [Figure 2C; ANOVA, *F*_(2, 15)_ = 48.5, *p* < 0.001]. Therefore, the rats actually modulated their behavior according to their expectation of a high probability of reward under the H-RP condition.

### Neurons for reward expectancy and task performance in amygdala and hippocampus

**Figure 3:**
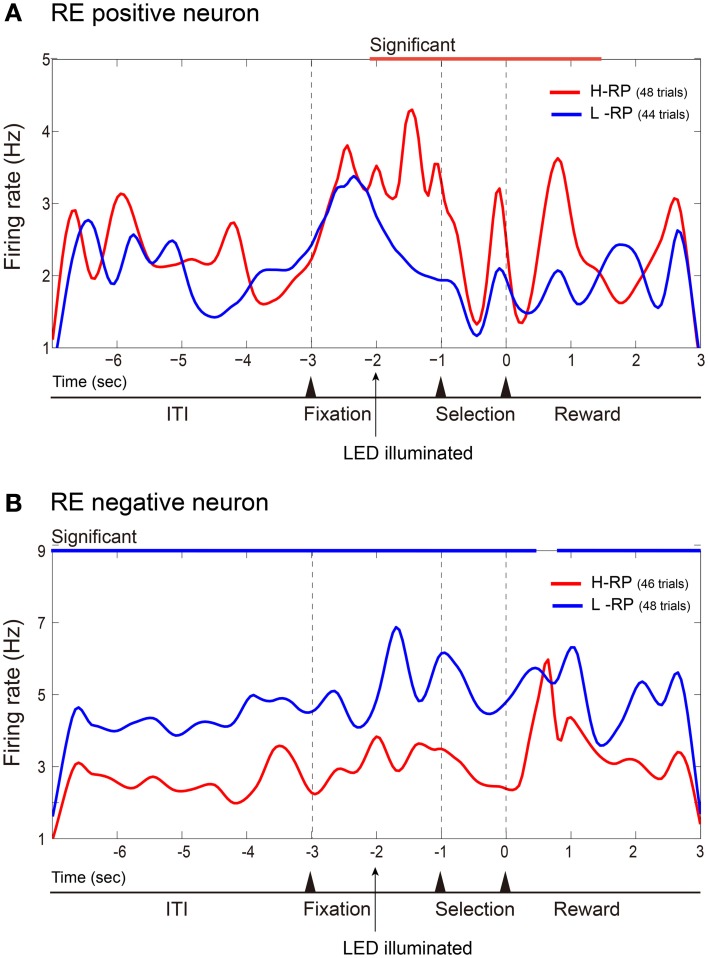
**Examples of amygdala RE neurons**. Firing rates in all correct trials of a session are cumulated and smoothed separately under the H-RP and L-RP conditions. **(A)** Example of RE positive neuron, which fired more frequently for the rat under the H-RP condition than the L-RP condition. The time of zero is the time when the rat poked its nose into the selected hole. The period of significantly (*p* < 0.05) higher firing rate under the H-RP condition is indicated by the red horizontal line at the top of the graph. **(B)** Example of RE negative neuron, which fired more frequently for the rat under the L-RP condition than the H-RP condition. Periods of significantly (*p* < 0.05) higher firing rates under the L-RP condition are indicated by the blue horizontal lines at the top of the graph.

Figure 3 shows averaged and smoothed firing-rate histograms of two examples of amygdala neurons recorded during the task. Both neurons changed their activities in accordance with the different RP conditions. The neuron in the upper portion (Figure 3A) gradually increased its firing rate during the fixation phase. This increase was sustained even after the fixation phase under the H-RP condition. Under the L-RP condition, this neuron decreased its firing rate after the presentation of the discriminative LED cue. Thus, the increase in firing rate was dependent on the rat's expectation of a high probability of reward. We classified such a neuron as an RE positive neuron. The amygdala neuron in the lower portion (Figure 3B) increased its firing rate under the L-RP condition during the task periods. We classified such a neuron as an RE negative neuron. In these examples of RE neurons, no change was observed in the firing rate for discriminative nose-pokes between the left and right holes.

Among 47 amygdala neurons recorded, 21 neurons were classified as RE neurons. The majority (17, 80.9%) of the RE neurons were RE positive neurons. Most RE neurons (18, 85.7%) also showed no significant difference in their firing rates between discriminative nose-pokes. Three amygdala neurons were classified as TP neurons, which showed differential activity between the discriminative nose-pokes. Two of the TP neurons also showed a significant firing-rate difference between RP conditions and could be classified as TP and RE neurons. Therefore, almost all of the task-related amygdala neurons were RE neurons.

**Figure 4:**
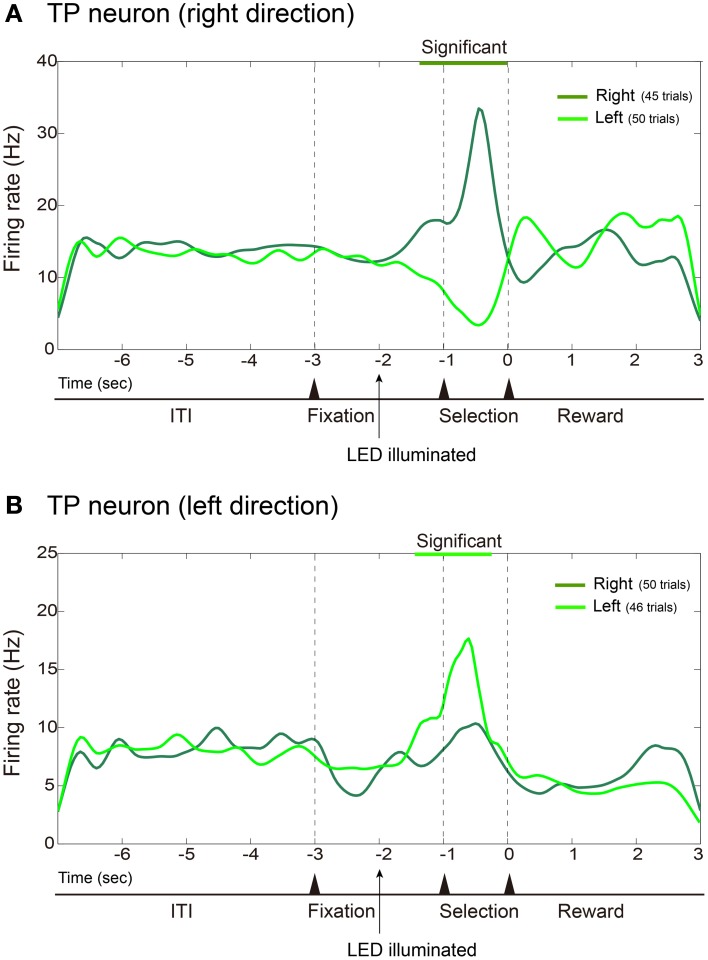
**Examples of hippocampal TP neurons**. Same format as Figure 3 except that firing rates are cumulated and smoothed separately when the rats poked their nose into the left or right hole. **(A)** Example of TP neuron that fired more frequently during the selection period prior to nose-pokes to the right hole. **(B)** Example of TP neuron that fired more frequently prior to nose-pokes to the left hole.

Figure 4 shows averaged and smoothed firing-rate histograms of two examples of hippocampal neurons recorded during the task. Each of the hippocampal neurons was a TP neuron that showed differential firing rates between discriminative nose-pokes to the left and right holes. The neuron in the upper portion (Figure 4A) increased its firing rate after the onset of the right discriminative cue and drastically increased it further in the selection period prior to nose-pokes to the right hole. In contrast, its firing rate gradually decreased after the onset of the left LED cue. Thus, the activity of this neuron depended on the performance of the discriminative behavior. The neuron in the lower portion (Figure 4B) is another example of a TP neuron. It increased its firing rate after the onset of the left LED cue and increased it further in the selection period prior to nose-pokes to the left hole. In these examples of TP neurons, firing rates between the RP conditions did not change.

Among 23 hippocampal neurons recorded, 10 neurons were classified as TP neurons and one neuron was classified as an RE neuron. Six of the TP neurons also showed significantly different firing rates between RP conditions, i.e., they exhibited selective firing for the cue directions and also changed their firing rates between the RP conditions. Thus, such TP neurons were also RE neurons. The one RE, not TP, neuron recorded from the hippocampus significantly increased its firing rate under the L-RP condition and thus was an RE negative neuron. Consequently, most of the task-related hippocampal neurons were TP neurons and the majority of them were also RE neurons that changed their firing rates in accordance with the differences in RP.

**Figure 5:**
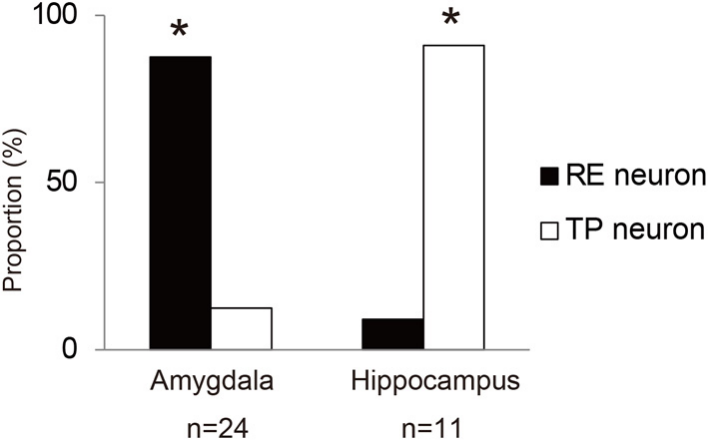
**Proportions of RE neurons and TP neurons in the amygdala and hippocampus**. The asterisks indicate a significant difference between RE and TP neurons.

To examine functional dissociation between the amygdala and the hippocampus, we used Fisher's exact test for the 2 × 2 contingency table of brain regions and task-related neurons (Figure 5). The results show that the amygdala has a significantly larger proportion of RE neurons and the hippocampus has a significantly larger proportion of TP neurons (*x*^2^ = 16.6463, *df* = 1, *p* < 0.001).

### Reward-expectation-modulated LFP coherence between amygdala and hippocampus

**Figure 6:**
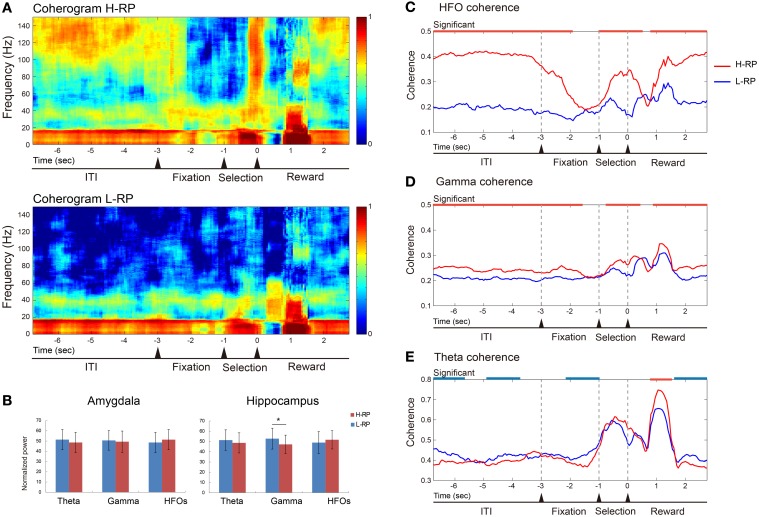
**Example of data of LFP coherence and power between the amygdala and the hippocampus. (A)** Coherence presented as coherograms under the H-RP (upper) and L-RP (lower) conditions. The ordinates indicate the frequency of coherence. The abscissas indicate the time course and the time of zero is the time when the rat poked its nose into the selected hole. Colors in the graph indicate the strength of coherence. **(B)** Averaged powers presented as normalized PSDs between H-RP and L-RP conditions in the amygdala (left) and hippocampus (right). Each of the three different bands of oscillation (theta, gamma, and HFOs) was compared. The asterisk indicates a significant difference between H-RP and L-RP. **(C)** HFO coherences between the amygdala and the hippocampus under H-RP and L-RP conditions. The ordinate indicates the strength of coherence. The abscissa indicates the time course and the time of zero means the time when the rat poked its nose into the selected hole. Periods of significantly (*p* < 0.05) stronger coherence under the H-RP condition are indicated by the red horizontal lines at the top of the graph. **(D)** Gamma coherences between the amygdala and the hippocampus under H-RP and L-RP conditions. Same format as **(C)**. **(E)** Theta coherences between the amygdala and the hippocampus under H-RP and L-RP conditions. Same format as **(C)**. Periods of significantly (*p* < 0.05) stronger coherence under the L-RP condition are indicated by the blue horizontal lines at the top of the graph.

Figure 6 shows an example of LFP coherences between the amygdala and the hippocampus recorded during the task. In the example, clear differences in the coherences between the different RP conditions in the upper and lower coherograms were observed (Figure 6A). We calculated averaged PSDs for the amygdala and hippocampus data under both RP conditions (Figure 6B). The normalized overall power in the amygdala did not differ between the RP conditions. Theta and HFOs power in the hippocampus showed no difference between the RP conditions, and only the normalized gamma power in the hippocampus under the H-RP condition was significantly lower than that under the L-RP condition [ANOVA, *F*_(1, 50)_ = 4.226, *p* < 0.05]. Therefore, the difference in LFP coherence between the RP conditions was not likely to result from an increase in the average PSDs.

As shown in Figure 6C, the HFO coherence was significantly higher under the H-RP condition during most task periods [ANOVA, *F*_(1, 6866)_ = 2428.3, *p* < 0.001]. This result indicates that the elevated HFO coherence of LFP was involved in a high expectation of reward in the rats, and this involvement was prominent during the ITI, selection period, and around and after reward delivery. The gamma coherence was significantly higher under the H-RP condition than under the L-RP condition during most of the task periods [*F*_(1, 6866)_ = 84.75, *p* < 0.001] (Figure 6D). This result shows that the elevated gamma coherence, similarly to the HFOs, was involved in the high expectation of reward in the rats, although the difference between the RP conditions was not as prominent as between the HFOs.

As shown in Figure 6E, the theta coherence under the H-RP condition significantly decreased in some periods of the task and was drastically ramped up during the period of reward delivery [*F*_(1, 6866)_ = 2387.29, *p* < 0.001]. This result indicates that, although the modulation of theta coherence under both RP conditions was similar, the theta coherence was somewhat involved in the low expectation of reward under the L-RP condition and prominently involved in getting a highly expected reward under the H-RP condition in the task.

### Trial-by-trial relationship between LFP coherence and behavioral performance

**Figure 7:**
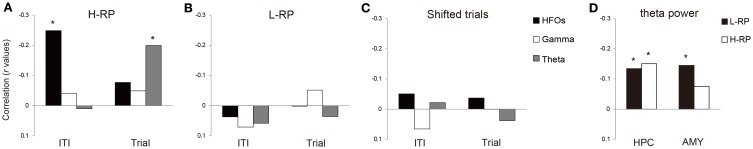
**Correlations between selection time and each of the three distinct bands of coherence (HFOs, gamma, and theta) during ITIs and trials. (A)** H-RP condition. The ordinate indicates the correlation presented as *r* values. The asterisks mean significantly stronger correlation among the three bands of coherence. **(B)** L-RP condition. Same format as **(A)**. **(C)** Shifted trials. Same format as **(A)**. **(D)** Correlations between selection time and theta power in the amygdala and hippocampus during trial periods.

We hypothesized that the activities of the amygdala and hippocampus would show synchrony of oscillation when the RE modulates the task performance. To test this hypothesis, we combined LFP data of all rats and examined the correlation between LFP coherence and behavioral performance, i.e., selection time (Figure 7). The correlation was examined during the ITI and trial periods.

Under the H-RP condition, coherent HFOs had a significant inverse correlation with selection time during the ITI (Figure 7A; *r* = −0.249, *p* < 0.001). During the trial periods, theta coherence showed a significant inverse correlation with selection time (Figure 7A; *r* = −0.200, *p* < 0.001). Such correlation, however, was not observed under the L-RP condition (Figure 7B). There was no correlation between LFP coherence and selection time in shifted trial periods (Figure 7C). These results show that when the rats highly expected a reward under the H-RP condition, LFP coherence had a relationship with the selection time of behavior, and the coherent HFOs during ITIs in particular had a predictive function of the subsequent behavioral performance.

To examine the possibility that the correlation between selection time and theta coherence simply reflected an increase in theta power associated with the movement speed during the trial periods, we calculated Pearson's *r* between the theta power during the trial periods and the selection time under both RP conditions. We found that the theta power of the hippocampus showed a significant inverse correlation under both RP conditions (Figure 7D; H-RP condition: *r* = −0.15, *p* < 0.001; L-RP condition: *r* = −0.134, *p* < 0.001). The theta power of the amygdala also showed a significant inverse correlation under the L-RP condition (Figure 7D; *r* = −0.145, *p* < 0.001). These results indicated that although the increase in theta power was clearly associated with the movement speed, the association did not correlate with the H-RP condition. Thus, the inverse correlation between task performance and theta coherence under the H-RP condition is not likely to be explained by an increase in theta power.

### Cross-frequency couplings of LFPs within and across amygdala and hippocampus

A recent study (Tort et al., 2013) reported interesting data suggesting that HFO power can be modulated by the theta phase in the hippocampus and neocortex, indicating the contribution of this theta-HFO coupling to cognitive processes such as memory. We hypothesize that such theta-HFO PAC is closely involved in the correlation between LFP coherence and behavioral performance and is important for modulating behavior on the basis of RE. Moreover, Tort et al. (2008) reported that cross-structure coupling between the striatal theta phase and the amplitude of the hippocampal HFOs occurs. We also hypothesize that such coupling interaction between the amygdala and the hippocampus occurs during ITI under the H-RP condition. To test these hypotheses, we examined MI within and across the amygdala and hippocampus during the ITI and trial periods. Three-way ANOVA showed that there was no main effect for all factors: REs (H-RP condition and L-RP condition), task periods (ITI and trial period), pairs for PAC (PACs within the amygdala, PACs within the hippocampus, amygdala theta phase—hippocampal HFO amplitude, and hippocampal theta phase—amygdala HFO amplitude). The interactions between REs and task periods, and among all factors were significant [*F*_(1, 17)_ = 9.734, *p* < 0.01; *F*_(3, 51)_ = 4.631, *p* < 0.01].

**Figure 8:**
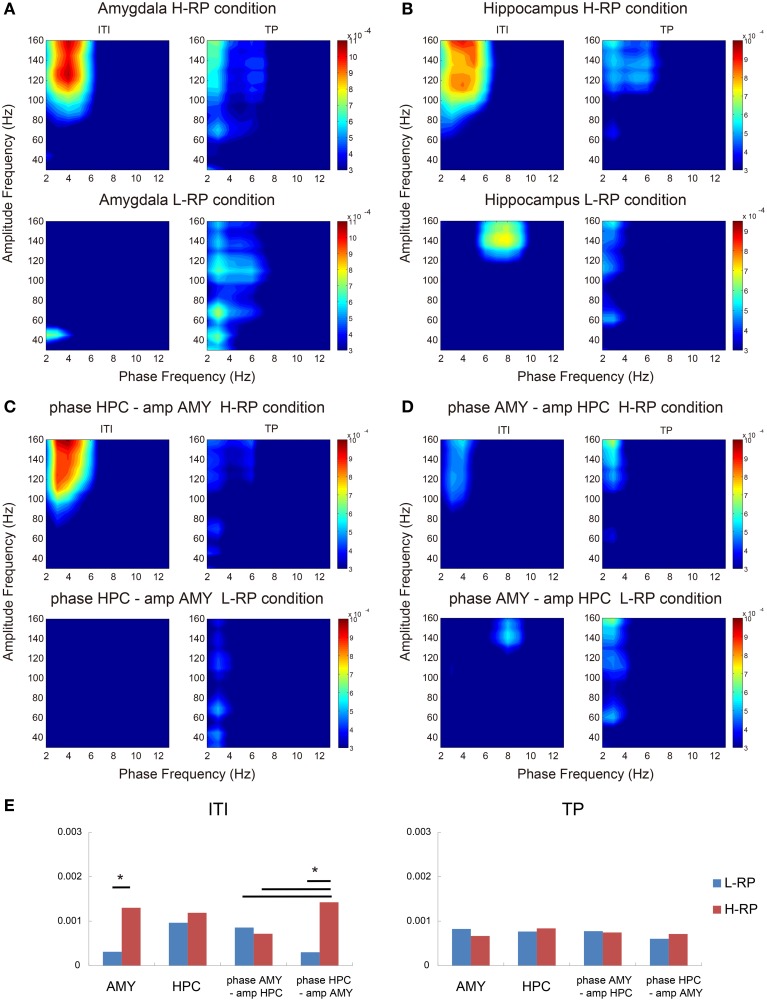
**Phase-to-amplitude mean comodulograms plotted for ITIs and trial periods. (A)** Within the amygdala under the H-RP (upper) and L-RP (lower) conditions. The ordinates indicate the ranges of frequency bands in fast oscillations modulated by the phase of slow oscillations. The abscissas indicate the ranges of frequency bands in slow oscillations modulating the amplitude of fast oscillations. Colors in the graph indicate the strength of MI. **(B)** Within the hippocampus. Same format as **(A)**. **(C)** Mean comodulograms in the hippocampal theta phase and the amygdala HFO amplitude. Same format as **(A)**. **(D)** Mean comodulograms in the amygdala theta phase and the hippocampal HFO amplitude. Same format as **(A)**. **(E)** Mean peak MIs of each comodulogram during ITIs (left graph) and the trial periods (right graph). Asterisks indicate significant differences of ^*^p < 0.05.

In both the amygdala and the hippocampus, theta-HFO PAC characteristically co-occurred during ITI (Figures 8A,B,E). Within the amygdala, MI in the theta phase and HFO power were significantly higher under the H-RP condition during ITI [Figures 8A,E; *F*_(1, 136)_ = 12.361, *p* < 0.001]. No such difference in peak MI, however, was observed within the hippocampus (Figures 8B,E). These results suggest that theta-HFO PAC within the amygdala emerges when the rats highly expect a reward, whereas PAC within the hippocampus emerges without a high expectation of reward. Note that the range of frequencies in which PAC occurs was different for different RP conditions in the hippocampus (Figure 8B), although PAC emerged during ITI. Specifically, theta-band oscillations under the H-RP condition modulated a wider range of HFO (>90 Hz), and the modulation tended to occur at low (3–6 Hz) theta frequencies. Interestingly, these ranges are similar to those of the amygdala under the H-RP condition. This result may suggest that the hippocampal PAC is influenced by the amygdala PAC, corresponding to the elevated HFO coherence of LFP (Figure 6C).

As shown in Figures 8C,D, cross-structure coupling did occur. The PAC between the hippocampus theta phase and the amplitude of amygdala HFOs during ITI under the H-RP condition was significantly higher than that under the L-RP condition [Figure 8C; *F*_(1, 136)_ = 16.002, *p* < 0.001] and the most prominent among the cross-structure couplings (Figure 8E; Ryan's method, *t* = 2.865, *p* < 0.05). In contrast, the PACs between the amygdala theta phase and the amplitude of hippocampal HFOs during ITI were not different for different RP conditions (Figures 8D,E). These results suggest that cross-structure couplings between the amygdala and the hippocampus are asymmetric and involved in the correlation between HFO coherence and behavioral performance.

## Discussion

### High expectation of future reward facilitates amygdala-hippocampus interaction via improved coherent oscillations

In the present study, we concluded that LFP coherence between the amygdala and the hippocampus improved under the H-RP condition when the rats highly expected the next reward (Figure 6). We confirmed that the rats indeed had a high expectation of reward under the H-RP condition by the behavior analysis (Figure 2). The coherence between the amygdala and the hippocampus also had a significant correlation with the subsequent behavioral performance (Figure 7). These findings are consistent with the notion that interactions between different brain regions are essential for modulating behavior on the basis of RE and that LFP oscillations contribute to such interactions. The finding that coherent HFOs in particular contributed to the regional interactions suggests that the short-time-scale communication between the amygdala and the hippocampus is coordinated with the oscillatory activity during the reward-expectation-induced behavioral modulation.

A notable finding of this study is that the different band coherences between the amygdala and the hippocampus showed different relationships with behavioral performance (Figure 7A). This finding suggests that different bands of oscillation have different roles in the amygdala-hippocampus interaction behind the adaptive behavior in accordance with reward expectancy. The theta coherence had a direct association with the selection time of behavior during trials. This band of coherence, therefore, may reflect the brain interaction for task performance. Supporting this assumption, some previous studies reported that hippocampal theta oscillation was correlated with the improvement of various task-related behavioral performances such as spatial navigation, memory-based behavior, and running speed (Buzsaki, 2005; Rutishauser et al., 2010; Ahmed and Mehta, 2012). On the other hand, the HFO coherence was predictively associated with the selection time under the H-RP condition, suggesting that this band of coherence may reflect the brain interaction for RE. By switching the different bands of frequency of their oscillatory interaction, these regions might change the type of information they communicate.

### Functional dissociation between the amygdala and the hippocampus

The types of task-related neurons depend on the brain regions in this study. We found more RE neurons in the amygdala and more task performing (TP) neurons in the hippocampus. These results suggest that the amygdala represents expectation of various reward probabilities and the hippocampus is involved in the appropriate behavioral performance required for the task. This suggestion is also supported by previous findings that the amygdala encodes the predictive representation of future reward (Schoenbaum et al., 1998; Belova et al., 2007; Savage and Ramos, 2009; Roesh et al., 2010), and the hippocampus has important roles in behavioral performance such as spatial navigation (Buzsaki, 2005) and in the improvement of task-related cognition and behavior (Rutishauser et al., 2010; Ahmed and Mehta, 2012).

A question that remains to be answered is what roles the oscillatory interaction between the amygdala and the hippocampus have in reward-expectation-induced behavioral modulation. One possibility is that the interaction serves to bind different information about reward and behavior, which are represented in the amygdala and hippocampus, respectively. Some groups previously studies reported that the interaction of theta waves between these regions contributed to the consolidation and retrieval of fear memory (Seidenbecher et al., 2003; Pape et al., 2005), and the extinction of fear was controlled by theta-rhythmic simultaneous electrical stimulation to these regions (Lesting et al., 2011). In addition to those studies, disconnection between the amygdala and the hippocampus was shown to impair reconsolidation of cocaine-related associative memory (Wells et al., 2011). In this study, the interaction between these regions might associate RP, which resulted in reward expectancy, with behavioral performance. The improved LFP coherence might correspond to the rapid retrieval or recall of such associative memory, hence improving the behavioral performance based on reward expectancy.

Another possibility is that the oscillatory interaction reflects the modulation of the hippocampal function by the amygdala. Firing properties or long-term potentiation of hippocampal cells can be altered by electrical stimulation of the amygdala (Ikegaya et al., 1994; Kim et al., 2012), and by reward information (Kobayashi et al., 1997; Holscher et al., 2003; Tabuchi et al., 2003; Hok et al., 2007; Kennedy and Shapiro, 2009; Singer and Frank, 2009; Dupret et al., 2010). Actually in the present study, many TP neurons in the hippocampus changed their firing rates for different reward probabilities. It is suggested, therefore, that the amygdala influences the firing rates and synchronization of hippocampal neuronal populations through the neuronal oscillations and that the improved LFP coherence corresponds to the strength of this modulation.

### Asymmetric cross-structure coupling between the amygdala and the hippocampus

We found cross-frequency couplings within and across the amygdala and hippocampus during ITI (Figure 8). The PAC within the amygdala varied considerably with the RP conditions, suggesting that cross-frequency couplings of theta-HFO in the amygdala are derived from RE in the rats. In contrast, PAC within the hippocampus emerges without expectation of reward, although the ranges of theta and HFO bands were different for different RP conditions (Figure 8B), and is involved in decision-making or behavioral choice (Tort et al., 2008). These findings suggest that cross-frequency couplings of theta-HFO in the hippocampus more strongly reflect task-performing-related cognitive processes. Therefore, the view of the functional dissociation between the amygdala and the hippocampus is also supported by the observations of cross-frequency coupling within these regions.

Moreover, the PAC between the hippocampus theta phase and the amplitude of amygdala HFOs during ITI under the H-RP condition was very distinct (Figure 8C). This asymmetric cross-structure coupling between the amygdala and the hippocampus might explain the elevated HFO coherence and the changes in the ranges of the hippocampal theta and HFO frequencies. First, the PAC within the amygdala under high expectation of reward allows the amygdala HFO to be modulated by the hippocampal theta phase. Herewith, the amygdala HFO influences the hippocampal HFO. Then, the range of the hippocampal HFO band changes and the PAC within the hippocampus becomes similar to that within the amygdala. From this view point, the elevated HFO coherence can reflect the process that the activities of the amygdala affect those of the hippocampus, and our observation of the oscillatory interaction between the amygdala and the hippocampus might reflect the information flow during the behavioral modulation based on RE. Future works are needed to confirm this hypothesis. Consequently, we conclude that the activities of the amygdala and hippocampus are synchronized in oscillation, which is one of the mechanisms of how RE modulates goal-directed behavior.

## Future prospects

Although we focused on the amygdala and hippocampus in this study, other brain regions, in particular, the prefrontal cortex (PFC), and the striatum, should be considered to play some important roles in behavioral modulation on the basis of RE (Corbit et al., 2001; Ostlund and Balleine, 2007; Holmes et al., 2010). The interaction between PFC, especially the orbitofrontal cortex, and the amygdala is crucial for generating and using the predictive representation of future reward (Schoenbaum et al., 2003; Holland and Gallagher, 2004; Schoenbaum and Roesch, 2005). In addition, the prelimbic area of the medial PFC might be involved in generating representations of reward in connection with the hippocampus (Hok et al., 2005; Burton et al., 2009), and the oscillation between PFC and the hippocampus is related to the regulation of goal-directed memory and behavior (Jones and Wilson, 2005; Benchenane et al., 2010). The striatum is projected from the amygdala and hippocampus and is reported to convert motivational signals to motor signals (Holmes et al., 2010; Retailleau et al., 2012). The interaction between the striatum and the hippocampus contributes to binding the representation of places to the representation of rewards (Ito et al., 2008; van der Meer and Redish, 2011). Moreover, coherent gamma oscillation between the striatum and the amygdala increases during the learning and expression of appetitive conditioned behaviors (Popescu et al., 2009). All of these findings suggest that extensive interaction among these brain regions is involved in the mechanisms of behavioral modulation based on RE. Research into oscillatory interactions among such broad networks will reveal the brain mechanisms behind reward-expectation-modulated goal-directed behavior.

### Conflict of interest statement

The authors declare that the research was conducted in the absence of any commercial or financial relationships that could be construed as a potential conflict of interest.
